# The importance of including the C-terminal domain of PTP1B_1-400_ to identify potential antidiabetic inhibitors

**DOI:** 10.1080/14756366.2023.2170369

**Published:** 2023-03-30

**Authors:** Andrea Coronell-Tovar, Francisco Cortés-Benítez, Martin González-Andrade

**Affiliations:** a Departamento de Bioquímica, Facultad de Medicina, Laboratorio de Biosensores y Modelaje molecular, Universidad Nacional Autónoma de México, Ciudad de México, México; b Departamento de Sistemas Biológicos, División de Ciencias Biológicas y de la Salud, Universidad Autónoma Metropolitana-Xochimilco (UAM-X), Ciudad de México, México

**Keywords:** Diabetes mellitus PTP1B_1-400_, kinetic assay, inhibidors PTP1B, docking PTP1B

## Abstract

In the present work, we studied the inhibitory and kinetic implications of classical PTP1B inhibitors (chlorogenic acid, ursolic acid, suramin) using three enzyme constructs (*h*PTP1B_1-285_, *h*PTP1B_1-321_, and *h*PTP1B_1-400_). The results indicate that the unstructured region of PTP1B (300-400 amino acids) is very important both to obtain optimal inhibitory results and propose classical inhibition mechanisms (competitive or non-competitive) through kinetic studies. The IC_50_ calculated for ursolic acid and suramin using *h*PTP1B_1-400_ are around four and three times lower to the short form of the enzyme, the complete form of PTP1B, the one found in the cytosol (*in vivo*). On the other hand, we highlight the studies of enzymatic kinetics using the *h*PTP1B_1-400_ to know the type of enzymatic inhibition and to be able to direct docking studies, where the unstructured region of the enzyme can be one more option for binding compounds with inhibitory activity.

## GRAPHICAL ABSTRACT

**Figure:**
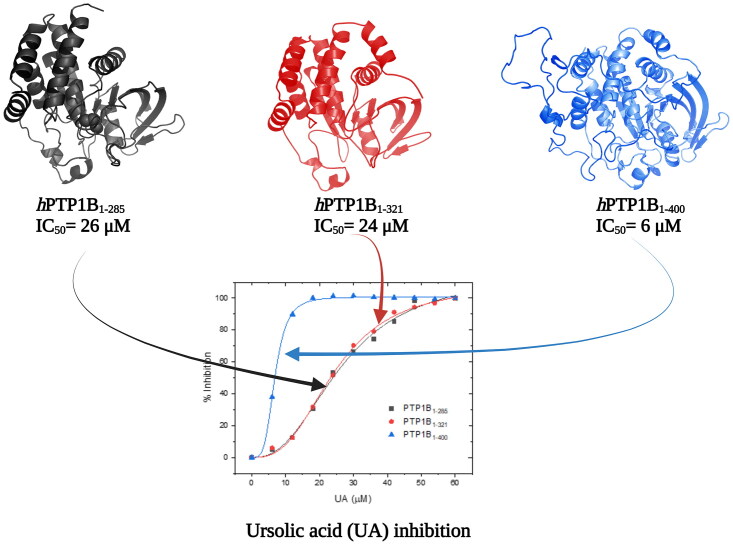

## Introduction

In recent years, the updated classification of diabetes mellitus (DM) published by the American Diabetes Association (ADA) in 2021 identifies four types of diabetes. (1) Type 1 diabetes (due to autoimmune-cell destruction, usually leading to absolute insulin deficiency, including latent autoimmune diabetes of adulthood), (2) Type 2 diabetes (due to a progressive loss of adequate b-cell insulin secretion frequently on the background of insulin resistance), (3) Specific types of diabetes due to other causes, and (4) Gestational diabetes mellitus (diabetes diagnosed in the second or third trimester of pregnancy that was not overt diabetes prior to gestation)^1^. The most critical clinical forms are type 1 DM (T1DM) and type 2 DM (T2DM). The latter is the type of diabetes for which between 90 and 95% of diagnosed cases of diabetes are registered worldwide. DM is a metabolic disorder characterised by hyperglycaemia, glycosuria, and some types of ketonaemia. These pathologies cause various complications, such as retinopathies, neuropathies, and peripheral vascular insufficiencies. The dramatic increase in DM in recent years may be related to multiple factors, including obesity and a sedentary lifestyle in the population^2^.

Due to the multifactorial nature of T2DM and the variety of molecular targets of the metabolic pathways associated with its aetiology, no specific pharmacological molecular target suggests a definitive treatment for this disease. However, various pharmacological targets for its treatment have been identified, such as dipeptidyl peptidase-4 enzyme (DPP-4), free fatty acid receptor 1 (FFAR1), G protein coupled receptors (GPCRs), α-Glucosidase, peroxisome proliferator-activated receptor (PPAR), augmenter of liver regeneration (ALR), G-protein coupled receptor for glucagon (GCGr), sodium-glucose linked transporter (SGLT), Phosphoenolpyruvate carboxykinase (PEPCK), and PTP1B, among the most important^2^.

### Protein tyrosine phosphatases (PTPs)

The phosphorylation/dephosphorylation of tyrosine residues is a mechanism for regulating the activity of proteins at the cellular level; it is essential for the normal functioning of signal transduction networks associated with the receptors of different hormones, cytokines and other bioactive molecules^3^. PTPs are divided into three subfamilies, classified as (1) Specific tyrosine phosphatases, (2) Dual-specific phosphatases, and (3) Low molecular weight phosphatases^4^. Several PTPs, such as protein tyrosine phosphatase α (PTPα), type receptor protein tyrosine phosphatase LAR (PTP-LAR), and PTP1B, have been implicated in the negative regulation of the insulin signalling pathway. The most convincing data implicate PTP1B as the most important regulator of this process^5^.

### Protein tyrosine phosphatases 1B (PTP1B)

**Figure 1.**
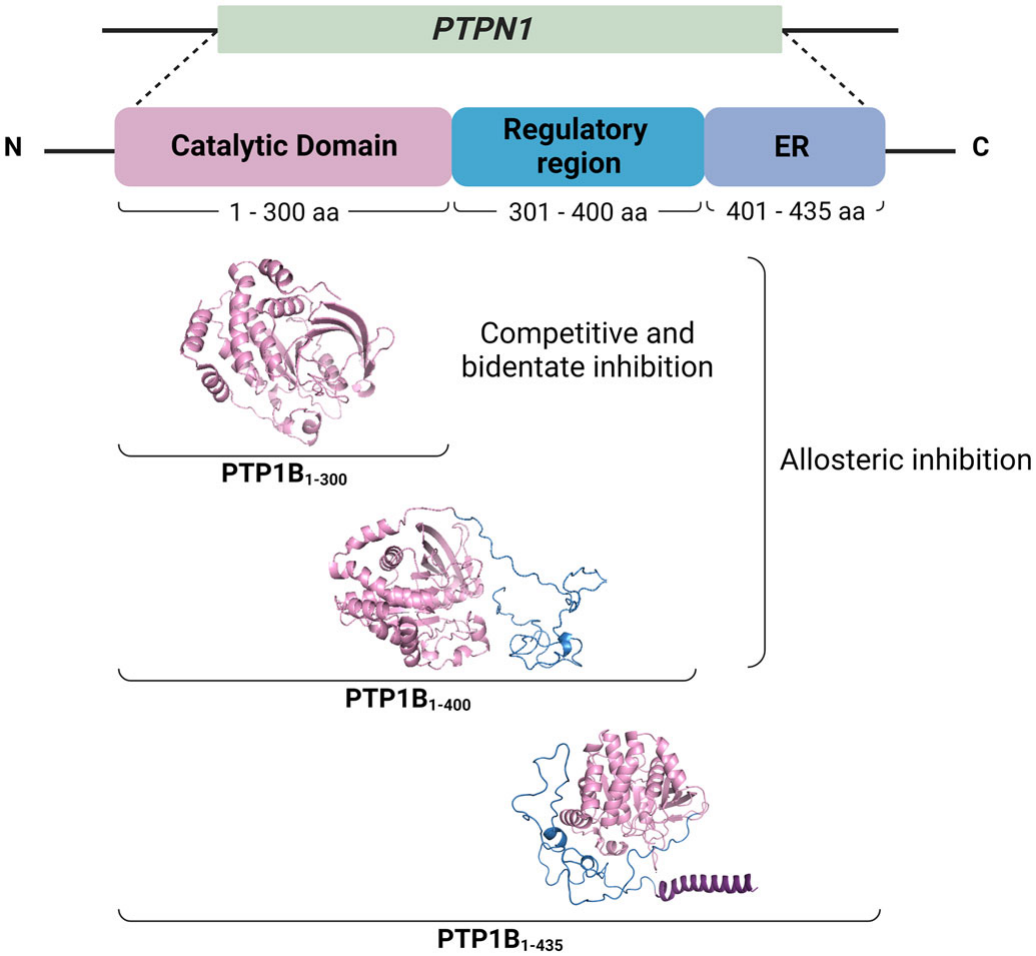
Structural domains of full-length PTP1B. The complete structure of *h*PTP1B is made up of an N-terminal catalytic domain (PTP1B_1-300_), an intrinsically disordered regulatory domain (PTP1B_301-400_), and a C-terminal ER localising domain (PTP1B_401-435_).

PTP1B is a monomeric cytosolic protein encoded by the *PTPN1* gene (locus 20q13), whose amino acid sequence includes 435 residues (∼50 kDa) distributed in three structural domains: a highly conserved N-terminal catalytic domain known as PTP domain (PTP1B_1-300_), an intrinsically disordered regulatory domain also known as proline-rich domain (PTP1B_301-400_), and a hydrophobic C-terminal signalling domain (PTP1B_401-435_) that directs the protein to the endoplasmic reticulum (ER)^6^. Knowledge of the structural domains of PTP1B has led to remarkable insights into the inhibition strategies targeted to the enzyme. On the one hand, the catalytic domain (PTP1B_1-300_) is the target site for developing competitive, bidentate and allosteric inhibitors. Finally, the regulatory domain (PTP1B_301-400_) is suggested as a second target site of allosteric inhibition. This second allosteric site is critical for interacting with potential inhibitors and enhancing their inhibitory potency^7^ (Figure 1).

Since the PTP1B catalytic domain has only been resolved by X-ray diffraction, the protein forms used in most enzymatic assays and computational studies are PTP1B_1-300_ and PTP1B_1-321_^8^. This is justified by the intracellular physiological loss of signalling residues 400-435 that directs the protein to the ER and by the intrinsically unstructured nature of the region that comprises residues 300–400 of the protein, which adopts an extended structure in solution^6^^,^^7^. However, PTP1B_1-400_ is the one found *in vivo*.

**Figure 2.**
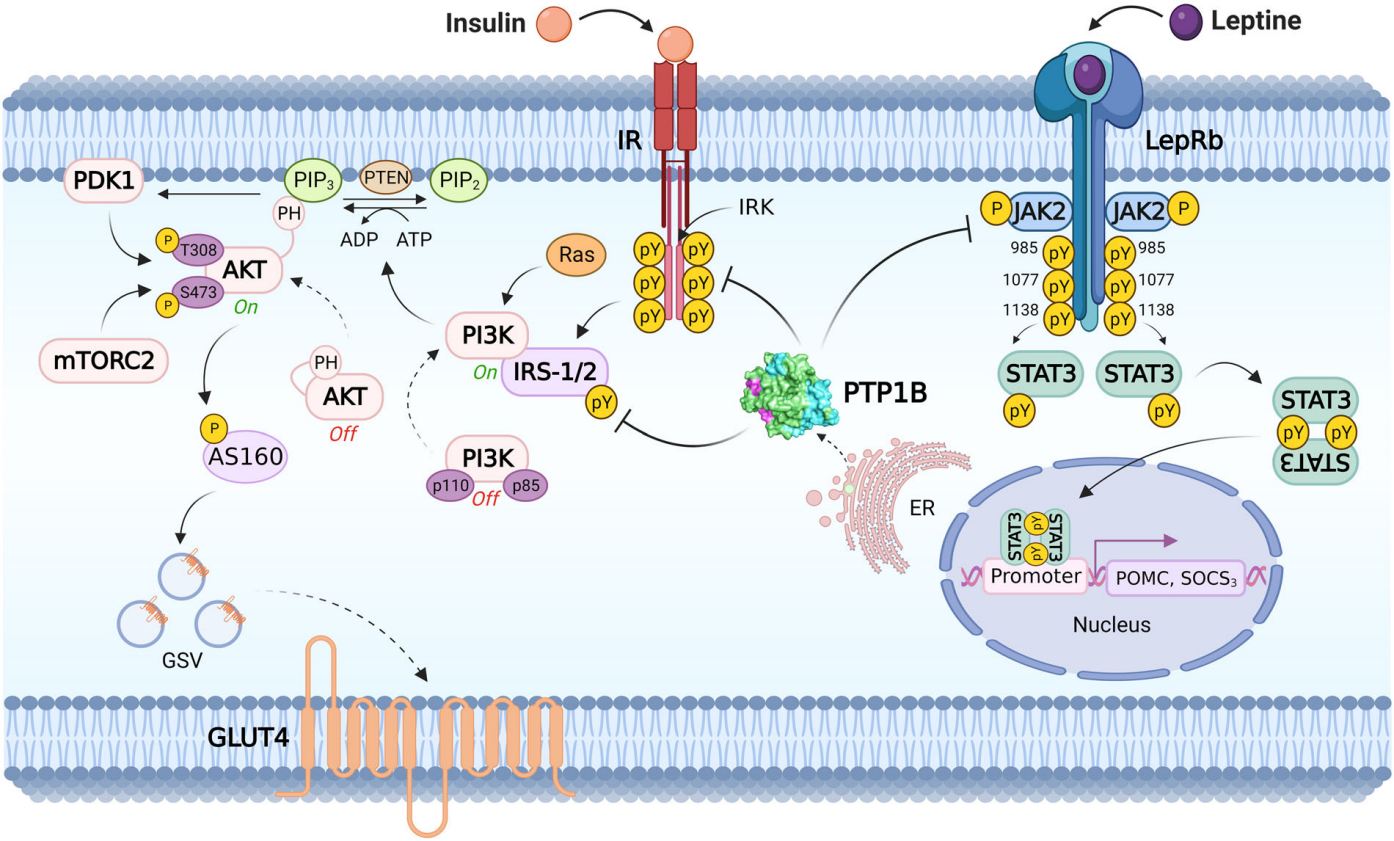
Main metabolic signalling pathways modulated by PTP1B. PTP1B acts as a negative regulator of insulin and leptin signalling pathways. In the insulin pathway, PTP1B dephosphorylates tyrosine residues in IR, IRS-1 and IRS-2, supporting glucose homeostasis by modulating GLUT4 transit. In the leptin signalling pathway, PTP1B dephosphorylates LepRb and JAK2, inactivating STAT3 and thus controlling the expression of genes POMC and SOCS_3_ involved in energy balance. *Abbreviatures*: ADP: adenosine diphosphate. ATP: adenosine triphosphate. Akt: Ak strain transforming. AS160: Akt substrate of 160 kDa. ER: endoplasmic reticulum. GLUT4: glucose transporter type 4. GSV: glucose storage vesicles. IR: insulin receptor. IRK: tyrosine kinase domain of the insulin receptor. IRS-1/2: insulin receptor substrate ½. JAK2: Janus kinase 2. LepRb: short isoform of the leptin receptor. mTORC2: mammalian/mechanistic target of rapamycin (mTOR) complex 2. PDK1: phosphatidylinositol-dependent kinase 1. PH: pleckstrin homology domain. PI3K: Phosphoinositide 3-kinases. PIP2: Phosphatidylinositol 4,5-bisphosphate. PIP3: phosphatidylinositol 3,4,5 trisphosphate. POMC: proopiomelanocortin. PTEN: phosphatase and tensin homolog. PTP1B: protein tyrosine phosphatase 1B. pY: phosphotyrosine. Ras: rat sarcoma virus. SOCS_3_: suppressor of cytokine signalling 3. STAT3: signal transducer and activator of transcription 3.

This phosphatase negatively regulates the insulin signalling pathway, promoting the dephosphorylation of phosphotyrosines (Tyr) in the β-subunit of the insulin receptor and probably in the IRS-1/2 protein^5^^,^^9^^,^^10^. In addition, this phosphatase also participates in the regulation of the leptin-dependent signalling pathway, and it is proposed that it is an essential regulator of the differentiation process of brown adipose tissue^5^^,^^11^ (Figure 2).

The insulin released from the pancreatic beta cell is transported into the cell, and it binds with its receptor producing the insulin-IR complex. Then, the intracellular part of the receptor is autophosphorylated, turning into an active kinase (sensitization). The abnormal signalling causes stimulation of PTP1B, which is involved in the desensitisation of the receptor by dephosphorylation. Therefore, inhibition of PTP1B has been considered as a potential drug target for the design of promising inhibitors. The effect of such inhibitors consists in delaying the receptor desensitisation, prolonging insulin effect for the treatment of diabetes II^12^.

Increased PTP1B activity in peripheral tissues appears essential for establishing insulin resistance, as suggested by numerous studies. This enzyme’s activity was increased in the muscle tissue of non-diabetic obese individuals suffering from insulin resistance^13^. On the other hand, inhibition of PTP1B expression by siRNA or gene deletion in laboratory mice increased their insulin sensitivity and improved glucose tolerance. In addition, it made them resistant to hypercaloric diet obesity^10^^,^^14^. Equally, it was shown that the inhibition of PTP1B expression in mice with polygenic insulin resistance (due to dysfunction of the insulin receptor and the IRS protein) improved glucose tolerance and insulin sensitivity and substantially decreased the incidence of diabetes^15^.

The background shows that PTP1B is a crucial protein in glucose homeostasis and is involved in the molecular mechanism of insulin resistance, a relevant condition in the pathogenesis of T2DM. Therefore, PTP1B can be an important therapeutic target in T2DM and other metabolic disorders such as obesity and metabolic syndrome^5^^,^^10^. Therefore, in recent years, there has been significant interest in the identification of PTP1B inhibitors, many of which have been identified from different sources and with various structural properties^7^^,^^16–22^. Additionally, current efforts include both *in vitro* and *in silico* experiments to study, design and discover new potent and selective inhibitors^23–26^.

There are some essential aspects in the search for PTP1B inhibitors; the most important are the potency, the type of inhibitor, and their selectivity. The first two can be performed using the appropriate enzyme form (*h*PTP1B_1-400_). Despite the fact that the last one is challenging due to the homology of the PTP domain among the PTPs family members, it can oppositely provide an opportunity to discover activators from related PTPs inhibitors^27^. However, selectivity can be achieved by performing comparatives activity assays with T-Cell Protein Tyrosine Phosphatase (TCPTP), which is the closest homologue of PTP1B. In this work, we study the enzymatic inhibition and the kinetic effect of three variants of *h*PTP1B (*h*PTP1B_1-285_, *h*PTP1B_1-321_, and *h*PTP1B_1-400_) with the inhibitors ursolic acid (UA), chlorogenic acid (CGA), suramin (SUR), sodium orthovanadate (SO), and Compound 5 b a derivative of 18β-glycyrrhetinic acid and docking studies to complement the experimental data of the enzyme-ligand complexes. The inhibitors used were chosen because some are classical inhibitors and are used as positive controls in many previous works and because they present different types of inhibition (competitive, mixed and acopetitive). The results indicate that the unstructured region of *h*PTP1B (300–400 aminoacid) is crucial to obtain reliable inhibition and kinetic parameters. On the other hand, kinetic studies are of great value in establishing the type of inhibition of bioactive compounds of PTP1B.

## Materials and methods

### Reagents

The full-length of human PTP1B (*h*PTP1B) coding gene sequence *PTPN1* (CCDS 13430.1; nucleotide ID: NM_002827.4; protein ID: NP_002818.1, 435 aa) codon optimised for *Escherichia coli* was obtained from OriGene (Rockville, MD, USA). The expression vector pET-28a(+) was obtained from Invitrogen (Waltham, MA, USA). *E. coli* strain DH5α and BL21 (DE3) were purchased from Invitrogen (Waltham, MA, USA). DNA extraction was performed with Kit QIAprep Spin Miniprep (Qiagen, Hilden, Germany). The forward-reverse pair primers for the expression of the three *h*PTP1B constructs, IPTG, imidazole, dithiothreitol (DTT), p-nitrophenyl phosphate (pNPP), chlorogenic acid (CGA), ursolic acid (UA), suramin (SUR), sodium orthovanadate (SO) were obtained from Sigma-Aldrich (St. Louis, MO, USA). Compound 5 b was synthesised from 18β-glycyrrhetinic acid according to the procedure of Ledy De-la-Cruz-Martínez and collaborators^28^.

### Construction of the coding genes for the three forms of hPTP1B

The three constructs of *h*PTP1B, including catalytic (represented by the short forms: *h*PTP1B_1-285_ and *h*PTP1B_1-321_) and C-terminal domain (*h*PTP1B_1-400_), were cloned by PCR from full-length *h*PTP1B coding gene (*PTPN1*) optimised for *E. coli* and inserted into a pET-28a(+) vector at the 5′*NdeI* and 3′*HindIII* restriction sites, resulting in a new construct pET-28a(+)-PTPN1. Three forward-reverse (Fwd-Rev) primer pairs were designed for each *h*PTP1B coding gen to construct so: Fwd 5′-TCATGGGTGATAGTTAAGTTCAGGACCAATG-3′ and Rev 5′- CATTGGTCCTGAACTTAACTATCACCCATGA-3′ to clone *h*PTP1B_1-285_, Fwd 5′- CTGGAACCGCATAATTGAAAGTGCCGTGAATTT-3′ and Rev 5′- AAATTCACGGCACTTTCAATTATGCGGTTCCAG-3′ to clone PTP1B_1-321_, and Fwd 5′- TGGTCATATGGAAATGGAAAAAG-3′ and Rev 5′-TGCAAGCTTAGTCTTCATCTTTTTC-3′ to clone *h*PTP1B_1-400_. The PCR amplification product size was confirmed by agarose gel electrophoresis. *E. coli* strain DH5α was transformed with all of the three PTP1B gene constructs. DNA concentration was determined by measuring the absorbance at 260 nm in a UV-Vis spectrophotometer (ThermoFisher, NanoDrop™ 2000/2000c). Finally PTP1B coding sequence gene construct was confirmed by DNA sequencing in the Unit of Molecular Biology, Institute of Cellular Physiology at UNAM (Mexico).

### Protein expression and purification

After DNA of each *h*PTP1B gene construct was sequenced (*h*PTP1B_1-285_, *h*PTP1B_1-321_, and *h*PTP1B_1-400_), they were transformed into *E. coli* strain BL21 (DE3). Transformed *E. coli* cells were grown in LB media (pH 7.5) containing kanamycin (30 mg/mL) at 37 °C for 6 h with continuous agitation (250 rpm). Once the cultures reached an *A*_600_ of 0.6 (about 6 h), protein expression was induced with 1 mM IPTG at 37 °C, 250 rpm for 6 h. After centrifuge (4500 rpm, 15 min, 4 °C), the grown and IPTG-induced cultures and bacterial pellet were resuspended in Tris buffer (pH 6.8) and lysed by sonication (10 cycles, intervals of 30 s) in an ice-water bath with an ultrasonic processor (Cole-Parmer, Vernon Hills, IL, USA). The cell lysate was centrifuged (13,000 rpm, 15 min, 4 °C), the supernatant was filtered with a PVDF membrane (pore size 0.45 µm) (Argos, Vernon Hills, IL, USA) and loaded onto a HisTrap HP immobilised metal affinity chromatography (IMAC) column (Cytiva, Marlborough, MA, USA). The column was equilibrated with 50 mM Tris, pH 6.8, with three column volumes. His-tagged protein was eluted with Tris 50 mM and 300 mM Imidazole with one column volume. PTP1B phosphatase activity of the collected fractions was confirmed by the pNPP activity assay (see below). Fractions containing *h*PTP1B were dialysed in 50 mM Tris, pH 6.8. The purity of the protein was followed by SDS-polyacrylamide gel electrophoresis (SDS-PAGE) using a 12% resolving gel.

### PTP1B activity assay

*h*PTP1B activity assays using para-nitrophenyl phosphate (pNPP) as substrate were performed to determine the intrinsic catalytic activity of all three *h*PTP1B constructs (*h*PTP1B_1-285_, *h*PTP1B_1-321_, and *h*PTP1B_1-400_) confirmed in the purification process, and the *in vitro* inhibitory effect of the tested compounds. Initial experiments were carried out to set up suitable conditions for the following enzyme activity assays. The optimum substrate and enzyme concentration were determined by testing different concentrations of each. pNPP activity assays were carried out in a final volume of 100 µL of Tris 50 mM, pH 6.8, containing purified *h*PTP1B 66 nM and pNPP 0.25 mM. Enzyme solutions containing pNPP and inhibitors were incubated at 37 °C for 15 min. The hydrolysis product’s absorbance, para-nitrophenol (pNP), was measured every 60 s for 30 min at 405 nm using a 96-well microplate absorbance reader (Accuris). Absorbance values were expressed in terms of molar product using a pNP extinction coefficient of 18,000 M^−1 ^cm^−1^^29^. The experiments were performed in triplicate. The variation of the molar pNP was analysed as a function of time, and the reaction rate was calculated as a function of pNPP concentration.

### hPTP1B inhibition assays

The inhibitory effect of ursolic acid (UA), chlorogenic acid (CGA), and suramin (SUR) on the three purified *h*PTP1B constructs (*h*PTP1B_1-285_, *h*PTP1B_1-321_, and *h*PTP1B_1-400_) were assessed at the previously established optimal conditions (final volume of 100 µL of Tris 50 mM, DTT 0.25 uM pH 6.8 at 37 °C for 15 min, absorbance lecture at 405 nm) using *h*PTP1B nM. IC_50_ assays were carried out in the assay buffer at fixed enzyme concentration (66 nM of each *h*PTP1B construct), pNPP 0.25 mM, and ten increasing concentrations of each inhibitor (diluted in 100% DMSO) as follows: UA in 5 µM increments (5 to 50 µM), CGA in 0.4 mM increments (0.4 to 4.0 mM), and SUR in 1.0 µM increments (1.0 to 10 µM). The negative control was prepared with the enzyme and substrate in the absence of an inhibitor. The experiments were performed in triplicate. The percentage of inhibition was plotted as a function of the inhibitor concentration. IC_50_ was calculated by regression analysis using Equation (1) (OriginPro 2018 (64-bit) SR1).

(1) $$\%\mathrm{PTP}1B=\frac{A_{100}}{1+{\left(\frac{i}{IC_{50}}\right)}^{s}}$$

where % *PTP*1*B* is the percentage of inhibition, *A_100_* is the maximum inhibition, *i* is the inhibitor concentration, *IC_50_* is the concentration required to inhibit the enzyme’s activity by 50%, and *s* is the cooperative degree.

### Enzyme kinetics of hPTP1B inhibition

In a similar way to the IC_50_, the assays to determine the enzyme kinetic parameters and inhibition mechanism were conducted in the same experimental conditions but varying concentrations of pNPP in 0.1 mM increments (0.1–0.5 mM), and using five increasing concentrations of each inhibitor according to previously determined IC_50_ values, as follows: UA 0, 1.5, 3.0, 4.5 and 9.0 µM; CGA 0, 0.5, 1.0, 1.6 and 3.0 mM; and SUR 0, 1.0, 2.0, 3.0 and 5.0 µM. The negative control was prepared with the enzyme and substrate in the absence of an inhibitor. The experiments were performed in triplicate. Enzyme inhibition kinetic parameters *K_m_* and *V_max_* were obtained by fitting data to the Michaelis-Menten model using Equation (2) (OriginPro 2018 (64-bit) SR1).

(2) $$y=\;\frac{V_{\max}\;x}{K_{m}\;+\;x},$$

*V_max_* is the maximum velocity, *x* is the inhibitor concentration, and *K_m_* is the Michaelis constant.

Fitting data determined PTP1B inhibition mechanisms to a nonlinear regression curve according to the equations defined for competitive, mixed, and uncompetitive inhibition models (Equations (3–5), respectively) (OriginPro 2018 (64-bit). SR1).

(3) $$y=\;\frac{V_{\max}\;(x)}{K_{m}\;\left(1+\left(\frac{I}{Ki}\right)\right)+x}$$

(4) $$y=\;\frac{V_{\max}\;(x)/\left(\left(1+I\right)/\left(\alpha \;K_{i}\right)\right)\;}{x+K_{m}\;\left(\left(1+I\right)/\;K_{i})/(1+I)/\left(\alpha \;K_{i}\right)\right)},$$

(5) $$y=\;\frac{V_{\max}\;(x)}{\;x+\left(K_{m}/(1+I)/K_{i}\right)}$$

*V_max_* is the maximum velocity, *x* is the substrate concentration, *I* is the inhibitor concentration, *K_m_* is the Michaelis constant, *K_i_* is the inhibition constant, and *α*=*V_max_* without inhibitor/*V_max_* with inhibitor.

### Docking

The structural model of the hPTP1B1-400 protein was obtained from AlphaFold Protein Structure Database developed by DeepMind and EMBL-EBI (https://alphafold.ebi.ac.uk/). The pdb file was downloaded from the following link: https://alphafold.ebi.ac.uk/entry/P18031^30^. This structure was subjected to a molecular dynamics simulation for 50 ns to find the most stable conformation of the unstructured region (301–400)^31^. The structures of the ligands were constructed and minimised using AVOGRADRO software^32^. AutoDockTools 1.5.4 was used to prepare the pdb files of *h*PTP1B_1-400_ and compounds. For stereoisomeric compounds, the conformer that presents greater stability after minimisation was coupled, maintaining the torsion angles detected by AutoDockTools. Polar hydrogen atoms and the Kollman united-atom partial charges were added to the protein structures. In contrast, Gasteiger-Marsili charges and rotatable groups were automatically assigned to the structures of the ligands. We use Autodock Vina to do the docking, covering the entire enzyme^33^. The grid box size was 76 × 56 × 60 Å in the x, y, and z dimensions and central coordinates of 42, 66, and 59 for x, y, z, respectively to the active site; 50 × 50 × 50 Å in the x, y, and z dimensions and central coordinates of 67, 80, and 79 for x, y, z, respectively to the allosteric site 1, and 76 × 56 × 60 Å in the x, y, and z dimensions and central coordinates of 75, 51, and 53 for x, y, z, respectively to the allosteric site 2 with exhaustiveness of 8. Visualising the best conformational states was achieved with PyMOL version 2.4.0 and Maestro version 5.3.156^34^.

## Results and discussion

### Recombinant hPTP1B_1-285_, hPTP1B_1-321_, and hPTP1B_1-400_

**Figure 3.**
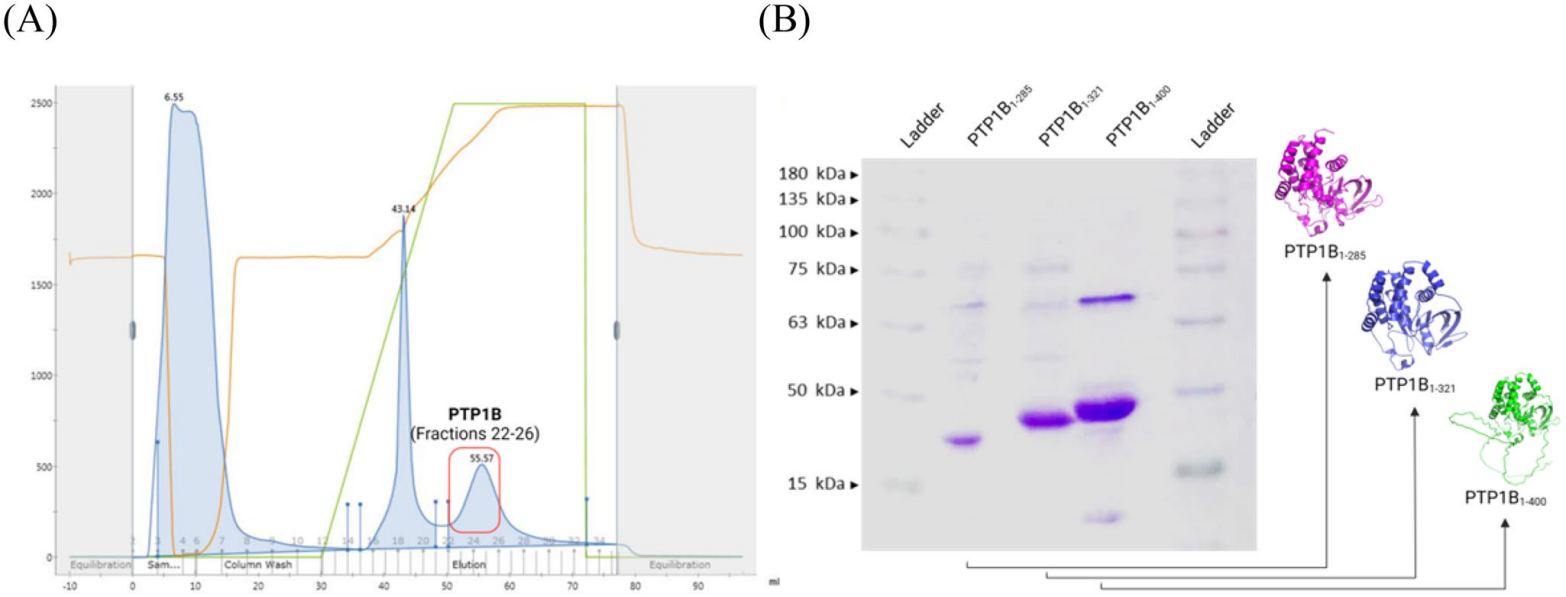
Recombinant *h*PTP1B purification process steps. (A) Fractions containing *h*PTP1B_1-400_ (22–26) collected during the HisTrap HP chromatography purification process. (B) SDS-PAGE (12%) electrophoresis gel band pattern shows the molecular weight of the three *h*PTP1B constructs: ∼33 kDa for *h*PTP1B_1-285_ (lane 2), ∼37 kDa for *h*PTP1B_1-321_ (lane 3), and ∼45 kDa for *h*PTP1B_1-400_ (lane 4).

*h*PTP1B is an attractive molecular target recently for developing and discovering bioactive antidiabetic and anticancer molecules. Inhibitory activity assays have been performed with different forms of the enzyme, where inhibitory activity has been reported and the latter may be underestimated depending on the *h*PTP1B segment used and the type of inhibition of the compound to be reported. We constructed three forms of *h*PTP1B (*h*PTP1B_1-285_, *h*PTP1B_1-321_, and *h*PTP1B_1-400_) to evaluate different types of inhibitors (competitive and non-competitive). *h*PTP1B_1-285_ and *h*PTP1B_1-321_ represent the short form of *h*PTP1B that includes the N-terminal catalytic domain. *h*PTP1B_1-400_ constitutes the long form of the protein that includes the intrinsically disordered C-terminal regulatory domain. Figure 3, shows the chromatogram of the purification by affinity chromatography and its follow-up by electrophoresis. The purified constructs showed a yield of 32, 40, and 56 mg per litre of culture medium for *h*PTP1B_1-285_, *h*PTP1B_1-321_, and *h*PTP1B_1-400_, respectively. The *K*_m_ of the sutrate (pNPP) is 16.37 ± 1.55, 15.66 ± 1.68, and 2.03 ± 0.11 mM for the *h*PTP1B_1-285_, *h*PTP1B_1-321_, and *h*PTP1B_1-400_, respectively.

### hPTP1B inhibition assays

**Figure 4.**
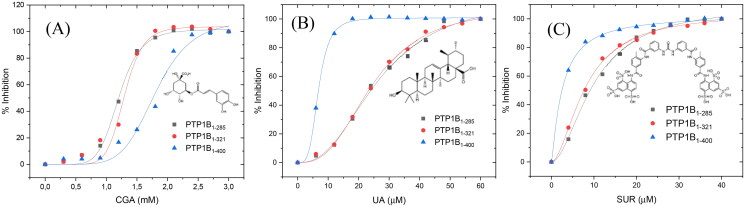
Inhibition assay using three constructs of the *h*PTP1B enzyme. (A) Chlorogenic acid (CGA). (B) Ursolic acid (UA). (C) Suramin (SUR). These inhibition assays were performed in a final volume of 100 μL of Tris 50 mM, DTT 0.25 uM, pH 6.8. *h*PTP1B constructs (*h*PTP1B_1-285_, *h*PTP1B_1-321_, and *h*PTP1B_1-400_) (66 nM) were incubated with ten increasing concentrations of each inhibitor at 37 °C for 15 minutes. Assay solutions (*h*PTP1B constructs + assay buffer) without inhibitors were used as a negative control. The absorbance was measured at 405 nm. Data are representative of three independent experiments.

Figure 4 shows the inhibition assays of classical inhibitors with the three different forms of *h*PTP1B. The IC_50_ observed for CGA is similar in all three enzyme forms. However, for the UA and SUR, the IC_50_ estimated using *h*PTP1B_1-400_ is around four and three times lower concerning the shorter forms (PTP1B_1-285_ and PTP1B_1-321_), respectively (Table 1). These results indicate that the IC_50_ varies depending on the length of the *h*PTP1B used to perform the activity assay (short or long). Therefore, using the enzyme in its complete form (including the unstructured part, *h*PTP1B_1-400_), which is the one found *in vivo*, has positive repercussions in calculating this parameter.

**Table 1.** Inhibition parameters of CGA, UA and SUR compounds using the three PTP1B constructs.

PTP1B form	CGA	UA	SUR
	IC_50_ (mM)	IC_50_ (µM)	IC_50_ (µM)
PTP1B_1-285_	1.20 ± 0.01	26.59 ± 1.46	9.95 ± 0.39
PTP1B_1-321_	1.29 ± 0.03	24.57 ± 0.66	8.00 ± 0.24
PTP1B_1-400_	1.80 ± 0.07	6.83 ± 0.07	2.59 ± 0.15

Many of the inhibitors reported in past years have used the short form of PTP1B to perform inhibition or kinetic assays. For example, N-aryl oxamic acid^35^, Ertiprotafib^20^, Celastrol^36^, use the *h*PTP1B_1-285._ 3-bromo-4,5-bis(2,3-dibromo-4,5-dihydroxybenzyl)-1,2-benzenediol (BDB)^21^, 2-(oxaloamino) benzoic acid (OBA)^37^, 2-(hydroxy-phenoxy) acetic acid^38^, Chlorogenic acid (CGA), Cichoric acid (CHA)^39^, Sodium orthovanadate (SO)^39–42^, use the *h*PTP1B_1-321_. In recent years the inhibitors Trodusquemine^7^^,^^43^, Ertiprotafib^18^, and Celastrol^36^ have reported inhibition parameters using both the short and complete forms of PTP1B (*h*PTP1B_1-321_ and *h*PTP1B_1-400_). For the Trodusquemine, the reported *K*_i_ are 4.0 and 0.6 µM for the short and long forms, respectively, about 6-fold lower with hPTP1B_1-405_. The same happens with celastrol, where IC_50_ are reported using PTP1B_1-298_ (IC_50_=4.8 µM) and PTP1B_1-393_ (IC_50_=2.1 µM), double the value using the short form.

Table 2 shows a compilation of previous reports of enzymatic assays of PTP1B inhibitors tested with the enzyme short and full-length constructs. We can see a variation in the parameters reported for the same compound depending on the PTP1B used for the test. On the other hand, the version found intracellularly is PTP1B_1-400,_ and the IC_50_ reported using this enzyme is lower, so inhibitors reported with the short version of PTP1B may have been overestimated.

**Table 2.** Previously reported inhibition and kinetic parameters using short and full PTP1B constructs.

Inhibitor	PTP1B structural region	IC_50_	*K_i_/K_m_/K_d_*	Inhibition type	Reference
N-aryl oxamic acid	PTP1B_1-288_	–	*K_i_* = 0.018 µM	Bidentate	^35^
Celastrol	PTP1B_1-298_	4.8 μM	*K_d_* = 6.1 μM	Non-competitive	^36^
	PTP1B_1-393_	2.1 μM	*–*		
Ursolic acid (UA)	PTP1B_1-298_	7.1 μM	*K_i_* = 25.8 μM	Non-competitive	^44^
	PTP1B_1-321_	6.6 μM	*–*	Competitive	^45^
	PTP1B_1-321_	3.08 μM	*–*	Competitive	^46^
	PTP1B_1-400_	5.6 μM	*K_i_* = 8.9 μM	Non-competitive	^28^
Ertiprotafib	PTP1B_1-299_	>20 μM	*–*	Nonclassic kinetics: Aggregation	^20^
	PTP1B_1-301_	*–*	*K_m_* = 555 µM	Nonclassic kinetics: Aggregation	^18^
	PTP1B_1-393_	*–*	*K_m_* = 568 µM		
Chlorogenic acid (CGA)	PTP1B_1-301_	*–*	*K_i_* = 8.2 mM *K_m_* = 3.3 mM	Non-competitive	^47^
	PTP1B_1-321_	9.82 μM	*–*	Non-competitive	^39^
Cichoric acid (CHA)	PTP1B_1-301_	*–*	*K_i_* = 1.4 mM *K_m_* = 2.8 mM	Competitive	^47^
	PTP1B_1-321_	10.51 μM	*–*	Non-competitive	^39^
BDB	PTP1B_1-321_	1.86 μM	1.1 μM	Competitive	^21^
DPM-1001	PTP1B_1-405_	0.10 μM	*K_d_* = 0.075 μM	Non-competitive	^48^
2-(Oxaloamino) benzoic acid (OBA)	PTP1B_1-321_	*–*	*K_i_* = 200 µM	Competitive	^37^
2-(Hydroxy-phenoxy) acetic acid	PTP1B_1-321_	*–*	*K_i_* = 9.0 µM	Competitive	^38^
1β,3α,19α-trihydroxy-2- oxours-12-en-28-oic acid	PTP1B_1-321_	13.18 μM	*K_i_* = 10.85 μM	Non-competitive	^49^
2β-hydroxy pomolic acid	PTP1B_1-321_	5.88 μM	*K_i_* = 7.56 μM	Mixed-competitive	^49^
18α-oleanolic acid	PTP1B_1-321_	10.50 μM	*K_i_* = 10.24 μM	Mixed-competitive	^49^
Sodium orthovanadate (SO)	PTP1B_1-321_	1.38 μM	*K_i_* =0.38 μM	Competitive	^39–42^
Suramin (SU)	PTP1B_1-321_	11.0 μM	*K_i_* = 4.0 μM	Competitive	^40^ ^,^ ^50^
	PTP1B_1-400_	4.1 μM	*K_i_* = 7.1 μM	Competitive	^28^
Trodusquemine	PTP1B_1-321_	*–*	*K_i_* = 4.0 µM	Non-competitive	^7^
	PTP1B_1-405_	*–*	*K_i_* = 0.6 µM		
	PTP1B_1-400_	1.0 μM	*–*	Non-competitive	^43^

### Enzyme kinetics of hPTP1B inhibition

**Figure 5.**
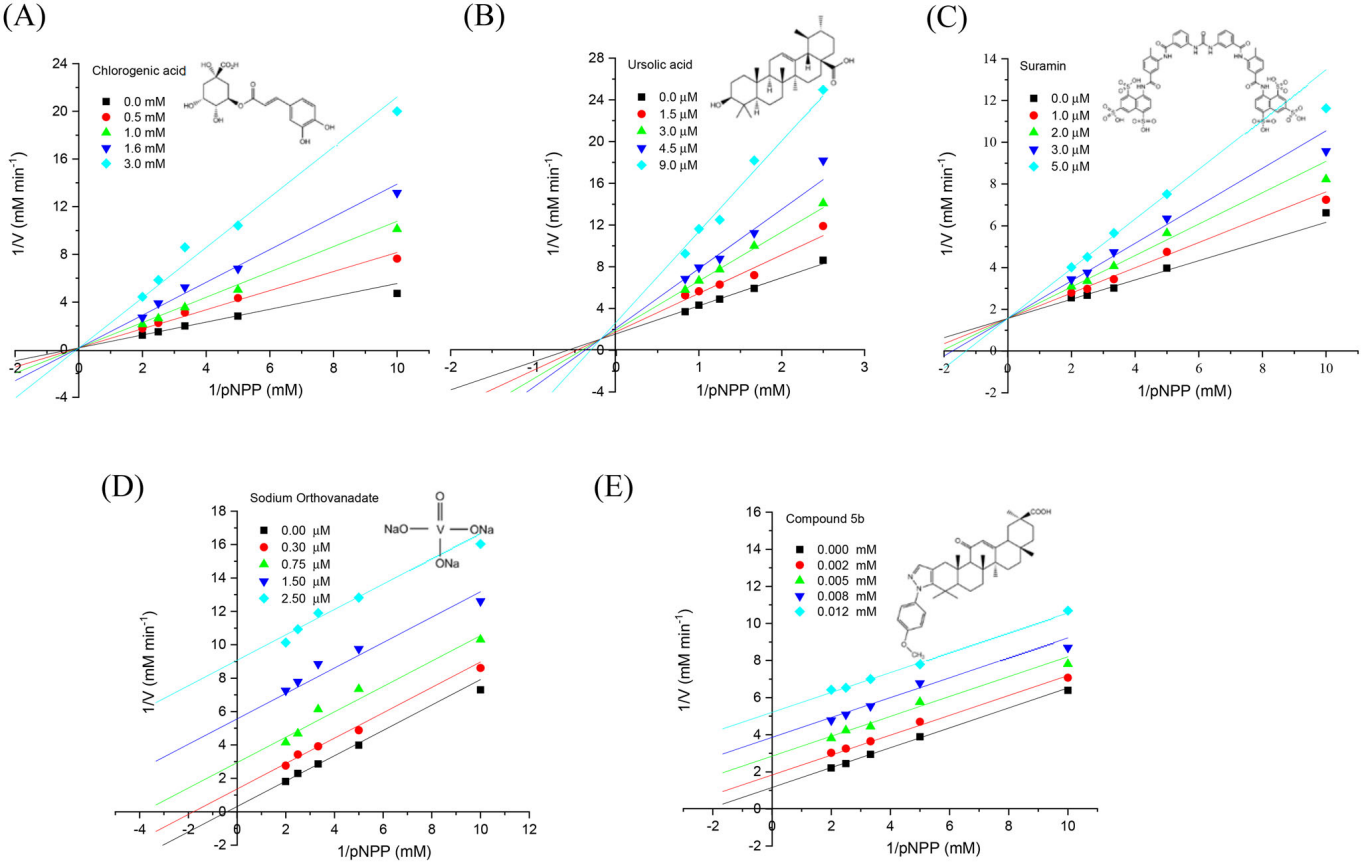
Enzyme kinetics of *h*PTP1B_1-400_ inhibition. Linweaver–Burk plots show the inhibition mechanism of tested compounds on *h*PTP1B_1-400_. (A) Chlorogenic acid. (B) Ursolic acid. (C) Suramin. (D) Sodium orthovanadate. (E) Glycyrrhetinic acid derivatives Compound 5b. The plots represent the reciprocal of the reaction velocity (1/V) as a function of the reciprocal of the pNPP concentration (1/[pNPP]). Data are representative of three independent experiments. The inhibitory mechanism for each compound was determined by fitting data to the equations defined for competitive, mixed, and uncompetitive inhibition models. Data represented in the graphs correspond to the best fit to each inhibition model (based on the *R*^2^ coefficient) (OriginPro 2018 (64-bit). SR1).

The kinetic parameters are fundamental since we can determine the affinity of an inhibitor for the enzyme and its possible mechanism of inhibition. With this information, we can conduct directed docking studies and obtain three-dimensional models of the enzyme-inhibitor complex. For example, Figure 5 and Table 3 show the results of kinetic studies of 5 inhibitors using *h*PTP1B_1-400_. For the CGA, the *K*_i_ is in the order of mM (1.03 mM), and the type of inhibition is competitive. Previous studies have reported non-competitive inhibition using *h*PTP1B_1-301_ and *h*PTP1B_1-321_^39^^,^^47^; this discrepancy may be due to the type of enzyme used for carrying out the studies. Based on our kinetic assay, the type of inhibition proposed for the UA was mixed in the order µM (*K*_i_ =4.0 µM). However, a competitive and non-competitive type of inhibition has been previously reported using *h*PTP1B_1-321_^45^ and PTP1B_1-298_^44^, respectively. For the SUR inhibitor, both previous studies^28^^,^^40^^,^^50^ and ours are in harmony when proposing a competitive inhibition, despite using different types of PTP1B. In the case of SO, the calculated *K*_i_ is in the nanomolar order (*K*_i_ =89 nM), and the type of inhibition was uncompetitive an order of magnitude higher *K*_i_ (0.38 µM) and a type of competitive inhibition have previously been reported for this inhibitor, using *h*PTP1B_1-321_. These discrepancies in the inhibition mechanisms reported by different authors may be due to the type of PTP1B used in the kinetic study or the statistical adjustments that are sometimes minimal. For example, for compound 5b a glycyrrhetinic acid derivative, the *R*^2^ are minimal for 4 different types of adjustment: competitive (*R*^2^=0.933), non-competitive (*R*^2^=0.978), uncompetitive (*R*^2^=0.996) and mixed (*R*^2^=0.975). On the other hand, kinetic studies allow us to direct computational theoretical studies such as docking since, knowing the type of inhibition, we can focus the conformational search for the inhibitor in particular regions of the enzymes.

**Table 3.** Kinetic parameters of inhibition and type of inhibition of different compounds using *h*PTP1B_1-400_.

Inhibitor	Type of inhibition	*V_m_*_ax_	*K*_m_	K_i_ (µM)
CGA	Competitive	6.71 ± 3.13	3.63 ± 1.88 mM	1.03 ± 0.07 mM
UA	Mixed	0.63 ± 0.08	1.70 ± 0.33 µM	4.03 ± 0.8 µM
SUR	Competitive	0.64 ± 0.02	0.29 ± 0.02 µM	3.0 ± 0. 2 µM
SO	Uncompetitive	3.20 ± 0.80	2.43 ± 0.70 nM	89.20 ± 0.225 nM
5 b	Uncompetitive	0.87 ± 0.03	0.46 ± 0.03 µM	3.42 ± 0.2 µM

### Docking studies

**Figure 6.**
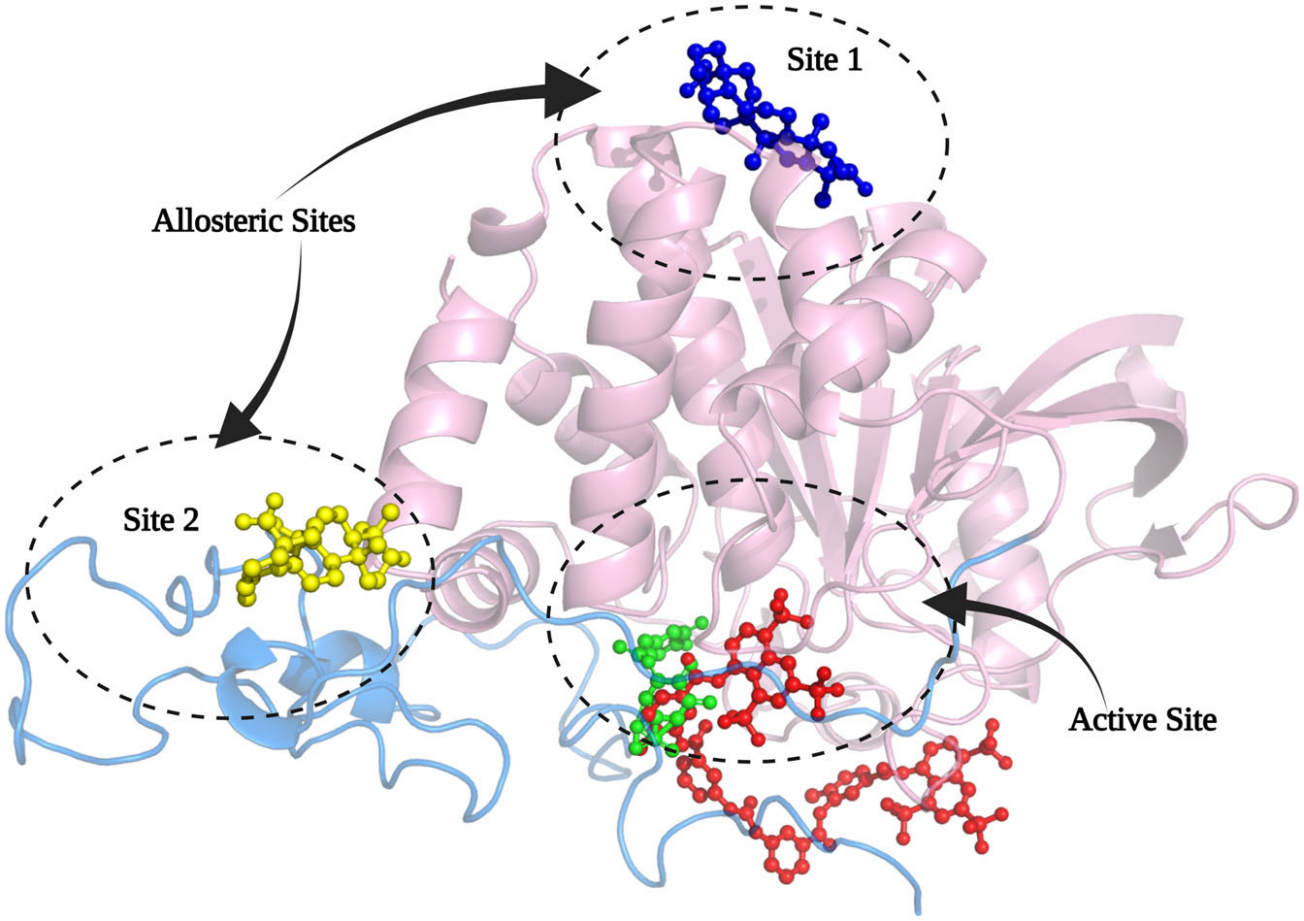
Structural model of PTP1B_1-400_ indicating the Active site and possible allosteric sites of inhibition. The catalytic domain is shown in pink cartoons and the unstructured region in a light blue cartoon, CGA in green sticks, SUR in red sticks, and UA in blue and yellow sticks.

**Figure 7.**
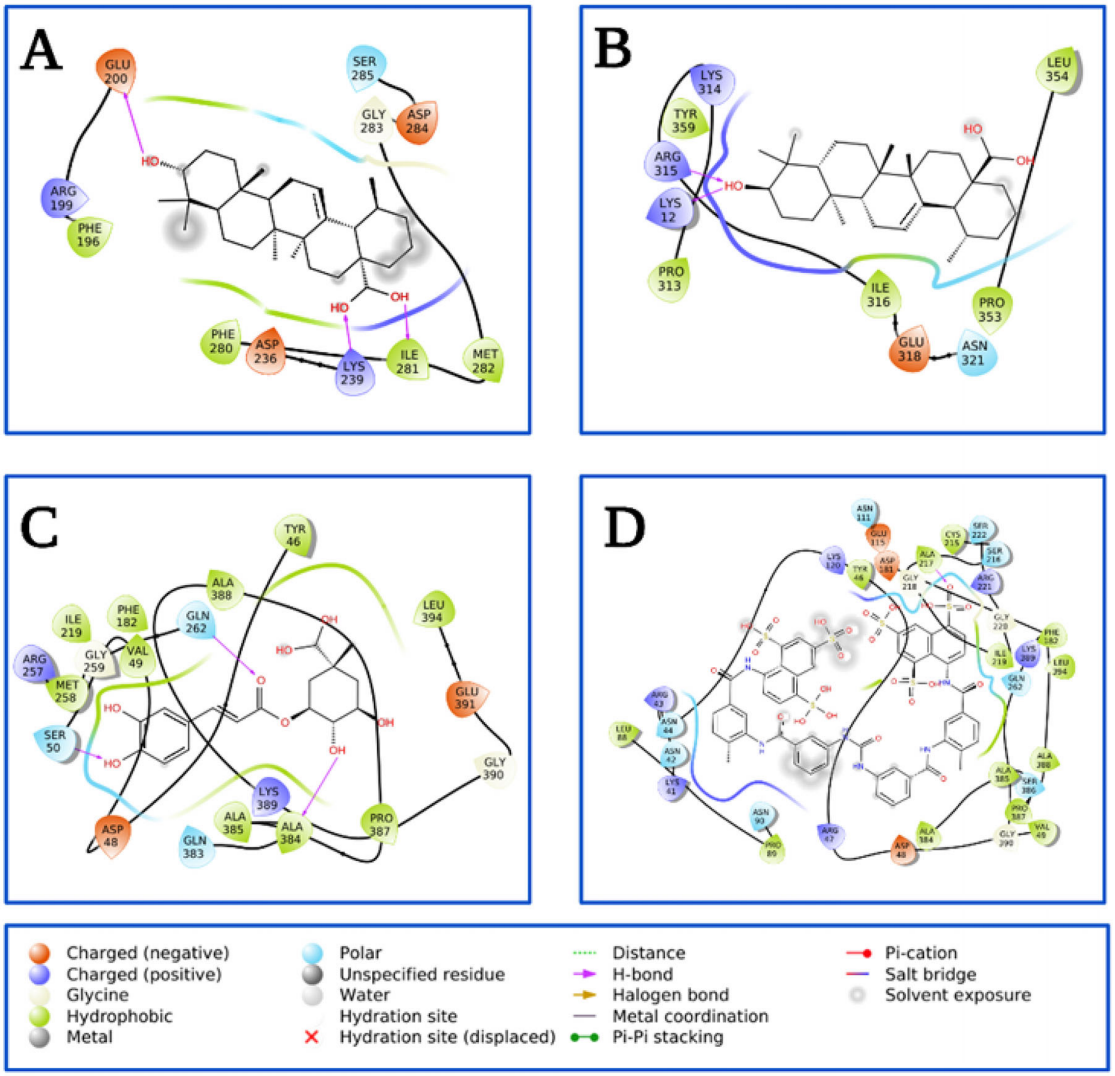
Analysis of the interacting amino acid residues in PTP1B-inhibitor complex. (A) UA-allosteric site 1, (B) UA-allosteric site 2, (C) CGA-catalytic site, and (D) SUR-catalytic site.PTP1B is shown in cartoons at the centre with compounds (**1**; orange stikers), (**2**; red stikers), (**3**; cyan stikers), (**4**; yellow stikers), and (**5**; green stikers). On the periphery are shown the analysis of the interactions with the residues at 4 Å.

Based on the information from the kinetic studies, we docked three inhibitors with *h*PTP1B_1-400_ (CGA, SUR and UA); the results are shown in Figures 6 and 7 and Table 4. CGA and SUR were directed to the catalytic site, and UA to the classically reported allosteric site and to the unstructured region where possible inhibitor-binding sites have also been recently reported^51^. Therefore, we have called site 1 the previously reported allosteric site and site 2 the one found in the unstructured region (Figure 6). The details at the molecular level of the inhibitors with PTP1B indicate hydrogen-bridge, hydrophobic, and ionic (negative and positive) interactions, among the most important (Figure 7). The theoretical binding parameters (*K*_i_ theoretical) are in the range of magnitude similar to the experimental values (Table 3). Two possible allosteric sites were studied for the UA, where the *K*_i_ were 22.23 and 4.75 µM, respectively. For the SUR and CGA inhibitors, the *K*_i_ theoretical were 23.98 and 747.12 µM, respectively. These studies are relevant to analyse specific interactions at the molecular level and propose modifications to the inhibitors to try to improve their inhibitory activity or specificity on *h*PTP1B.

**Table 4.** Theoretical binding properties of *h*PTP1B_1-400_ inhibitor complex.

Compound	*K*_i_ (µM)	EB (kcal/mol)	Interacting residues
UA site 1	22.23	−6.35	Phe196, Arg199, Glu200, Asp236, Lys239, Phe20, Ile281, Met282, Gly283, Asp284, Ser285
UA site 2	4.75	_7.26	Lys12, Pro313, Lys314, Arg315, Ile316, Glu318, Asn321, Pro353, Leu354, Tyr359
CGA	747.12	−4.25	Tyr46, Asp48, Val49, Ser50, Phe182, Ile219, Arg257, Met258, Gly259, Gln262, Gln383, Ala384, Ala385, Pro387, Ala388, Lys389, Gly390, Glu391, Leu394
SUR	23.98	−6.28	Lys41, Asn42, Arg43, Asn44, Tyr46, Arg47, Asp48, Val49, Leu88, Pro89, Asn90, Asn111, Glu115, Lys120, Asp181, Phe182, Cys215, Ser216, Ala217, Gly218, Ile219, Gly220, Arg221, Ser222, Gln262, Ala384, Ala385, Ser386, Pro387, Ala388, Lys389, Gly390, Leu394

## Conclusions

This work highlights the importance of using *h*PTP1B_1-400_ in inhibition and kinetic assays. The IC_50_ of the compounds CGA, UA, and SUR estimate using the three constructions of the enzyme (*h*PTP1B_1-285_, *h*PTP1B_1-321_, and *h*PTP1B_1-400_), indicates that this parameter using the complete enzyme is lower for the inhibitors AU and SUR. Therefore the IC_50_ of previously reported inhibitors that used the short forms of PTP1B may be underestimated. The complete form of *h*PTP1B is the one that is found *in vivo* in the cytosolic space, and it seems that the unstructured region plays an important role in the modulation of the enzyme’s activity. Another critical aspect of PTP1B inhibitors is knowing their inhibition mechanism, which can be known indirectly using enzyme kinetics studies, which can additionally be used to guide docking studies or molecular dynamics simulation. Molecular modelling studies can help improve known PTP1B inhibitors by analysing interactions at the molecular level of enzyme-inhibitor complexes and proposing synthetic derivatives. Based on the results obtained in this work, we can highlight some relevant points, such as (1) The importance of using the *h*PTP1B_1-400_ enzyme to get binding and kinetic parameters. (2) Carry out kinetic studies of the inhibitors to describe their mechanism of action. (3) Conduct in silico studies based on experimental studies, and (4) Redesign non-competitive inhibitors with low *K*_i_ (high potency) to favour selectivity and potency for PTP1B.
